# Emerging roles of primary cilia in the pathogenesis of amyotrophic lateral sclerosis

**DOI:** 10.3389/fnins.2025.1688839

**Published:** 2025-10-06

**Authors:** Hisashi Takahashi, Takashi Kasai, Aya Miyagawa-Hayashino, Tomoyuki Ohara

**Affiliations:** ^1^Department of Neurology, Kyoto Prefectural University of Medicine, Kyoto, Japan; ^2^Department of Pathology and Applied Biology, Graduate School of Medicine, Kyoto Prefectural University of Medicine, Kyoto, Japan; ^3^Department of Neurology, North Medical Center, Kyoto Prefectural University of Medicine, Yosano, Japan

**Keywords:** amyotrophic lateral sclerosis, primary cilia, ciliopathy, aging, NEK1, C21orf2, C9orf72, neurodegeneration

## Abstract

Amyotrophic lateral sclerosis (ALS) is a progressive neurodegenerative disease primarily affecting motor neurons, for which effective disease-modifying therapies remain elusive. Primary cilia are solitary microtubule-based organelles critical for signal transduction and have recently been implicated in ALS pathogenesis. In this review, we provide a basic overview of the structure, dynamics, and functions of primary cilia, particularly in the brain. We highlight accumulating evidence from ALS models showing altered ciliary structure and function and explore how mutations in ALS-associated genes such as *NEK1*, *C21orf2*, and *C9orf72* disrupt ciliogenesis and ciliary signaling. Moreover, we examine the interplays between primary cilia dysfunction and known ALS-related mechanisms, including loss of proteostasis, abnormal RNA metabolism, microtubule dysfunction, neuroinflammation, and mitochondrial dysfunction. Collectively, the evidence suggests a bidirectional relationship in which ciliary impairment and ALS pathomechanisms reinforce one another in a vicious cycle. We further discuss emerging therapeutic strategies targeting ciliary function, as well as the potential for primary cilia as novel clinical applications. Our review highlights primary cilia as a previously underappreciated yet potentially important component of ALS biology, offering novel insights into disease mechanisms and future therapeutic development.

## Introduction

Amyotrophic lateral sclerosis (ALS) is a progressive neurodegenerative disorder that primarily affects upper and lower motor neurons, leading to limb muscle weakness, swallowing difficulty, and eventually even respiratory failure. Paralysis typically begins focally and gradually spreads to other body regions, but the rate and pattern of progression vary considerably among patients. Some cases of ALS are known to be accompanied by frontotemporal lobar degeneration and the two are often considered part of the same disease spectrum. ALS has an annual incidence of approximately 1–2 cases per 100,000 person-years and a prevalence of about 4–6 per 100,000 individuals. Epidemiologically, incidence is slightly higher in males than in females, although this difference diminishes with increasing age. The typical age at onset ranges from 40 to 60 years, with a median survival of 2–3 years after symptom onset.

The neuropathological hallmark of ALS is cytoplasmic inclusions labeled by phosphorylated transactive response DNA-binding protein 43 (TDP-43), and the degeneration of motor neurons. Such degeneration is observed both in lower motor neurons, including anterior horn cells of the spinal cord and motor nuclei in the brainstem and in upper motor neurons in the precentral gyrus. TDP-43 pathology can remain restricted to these motor neurons, but in some cases it extends to other regions such as the inferior olivary nucleus, basal ganglia, and hippocampal dentate gyrus (Brettschneider et al., 2013).

While most ALS cases are sporadic, approximately 10%–15% are familial and associated with mutations in genes such as *superoxide dismutase 1 (SOD1)*, *TAR DNA-binding protein (TARDBP)*, *fused in sarcoma (FUS)*, and *chromosome 9 open reading frame 72 (C9orf72)* (Wang et al., 2023). Advances in genomic analysis have led to the identification of additional genetic contributors (Kenna et al., 2016; van Rheenen et al., 2016). Recent studies have highlighted multiple pathomechanisms, including non-cell-autonomous mechanisms involving glial cells, disturbances in RNA metabolism, neuroinflammation, cytoskeletal abnormalities, impaired proteostasis, and mitochondrial dysfunction (Hu et al., 2024). Despite extensive research, effective disease-modifying therapies remain limited, and the mechanisms underlying ALS pathogenesis thus urgently need to be identified.

In recent years, increasing attention has been directed toward the role of primary cilia in the pathogenesis of neurodegenerative diseases ( Álvarez-Satta et al., 2019; Ma et al., 2022; Ki et al., 2021). Primary cilia are solitary, microtubule-based projections found on the surface of most eukaryotic cells (Mill et al., 2023). While the existence of primary cilia has been recognized for decades, their significance came into focus in the early 2000s, particularly after the discovery of their role as key regulators in Sonic Hedgehog (Shh) signaling. Since then, primary cilia have been implicated in numerous physiological and pathological processes (Silva and Cavadas, 2023). Disorders arising from defects in ciliary structure or function are collectively referred to as ciliopathies. Initially, ciliopathy was a concept applied mainly to congenital disorders caused by mutations in genes encoding the structural components of cilia. However, growing evidence has expanded this concept to include aging and adult-onset diseases, in which primary cilia also play important roles. Ciliary dysfunction has now been reported in a wide range of age-related disorders, including cancer, diabetes, obesity, osteoarthritis, cardiovascular diseases, and sarcopenia. Furthermore, in age-associated neurodegenerative diseases such as Alzheimer’s disease and Parkinson’s disease, primary cilia and their functional impairments have been linked to disease mechanisms.

Although the relationship between primary cilia and ALS received only limited attention until recently, several pioneering studies have reported alterations in the structure of primary cilia and ciliary signaling in ALS model animals and patient tissues (Ma et al., 2011; Nakamura et al., 2008). More recently, mutations in several ALS-associated genes have been shown to disrupt ciliogenesis or ciliary function, suggesting a mechanistic link between these two fields (Tang et al., 2023; Noh et al., 2025; De Decker et al., 2025). As the understanding of both ALS pathophysiology and primary cilia biology deepens, overlapping mechanisms and shared cellular processes are beginning to be recognized. Accumulating evidence has led to a growing recognition that primary cilia dysfunction may contribute significantly to the pathogenesis of ALS, a concept that had previously been underappreciated.

In this review, we first provide an overview of the structure and dynamics of primary cilia, and related ciliary signaling. We then introduce recent observations regarding the involvement of ALS-related genes such as NIMA-related kinase 1 (NEK1), chromosome 21 open reading frame 2 (C21orf2), and C9orf72 in ciliogenesis. Next, we discuss the roles of ciliary signaling pathways, especially Shh signaling, transforming growth factor (TGF)-β signaling, and Wnt signaling in ALS. We further examine how primary cilia dysfunction may contribute to ALS pathogenesis, drawing upon relevant cellular and molecular studies. Finally, we highlight the potential for cilia-targeted therapeutic strategies in ALS and discuss current challenges and future directions in this emerging field. In contrast to prior reviews ( Álvarez-Satta et al., 2019; Ma et al., 2022; Ki et al., 2021), this article specifically focuses on ALS, providing an up-to-date synthesis of recent genetic evidence and highlighting the emerging roles of primary cilia in ALS pathomechanisms.

## Structure, dynamics, and functions of primary cilia

This section covers the basic structure and function of primary cilia as essential background for understanding their relevance to ALS. For further understanding, several prominent reviews are available (Mill et al., 2023; Gopalakrishnan et al., 2023; Macarelli et al., 2023; Nishimura et al., 2019; Anvarian et al., 2019). These reviews provide in-depth coverage of the basic biology of primary cilia, including their structure, dynamics, and signaling roles, whereas our article builds on this foundation to specifically examine their emerging involvement in ALS.

### Basic structure of primary cilia

**FIGURE 1:**
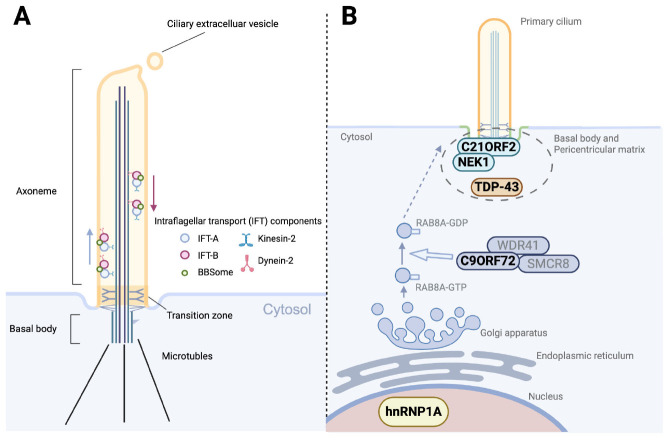
Structure of the primary cilium and ALS-related dysregulation of ciliogenesis. **(A)** Structural organization of the primary cilium. The axoneme, extending from the basal body, consists of doublet microtubules and is compartmentalized by the transition zone. Bidirectional intraflagellar transport (IFT), mediated by kinesin-2 (anterograde) and dynein-2 (retrograde), carries IFT-A, IFT-B, and BBSome protein complexes along the axoneme. Ciliary extracellular vesicles are released from the ciliary tip, contributing to intercellular communication and regulation of ciliary composition. **(B)** ALS-related genes and RNA-binding proteins implicated in ciliogenesis and vesicle trafficking. NEK1 and C21ORF2 localize to the basal body and pericentriolar matrix, where they regulate ciliary assembly and stability. TDP-43, a hallmark protein in ALS pathology, is also detected at the pericentriolar matrix and associates with other ALS-related RNAs and proteins. The C9ORF72–SMCR8–WDR41 complex functions as a GTPase-activating protein (GAP) for RAB8A, thereby downregulating RAB8A activity and restricting vesicle trafficking to the ciliary base. Since RAB8A activity normally promotes the membrane delivery required for ciliogenesis, this negative regulation by C9ORF72 contributes to the suppression of ciliogenesis. In addition, the ALS-associated hnRNPA1 D262V mutation induces widespread splicing alterations, many of which affect genes involved in ciliogenesis.

A primary cilium comprises several distinct subdomains (Figure 1A). At its core is the axoneme, a microtubule-based scaffold arranged in a characteristic “9 + 0” pattern of nine outer microtubule doublets without a central pair, extending from the basal body at the cell surface (Mill et al., 2023). The basal body, derived from the mother centriole, anchors the cilium to the cell and plays a central role in initiating cilium formation. The transition zone is located at the base of ciliary axoneme and acts as a selective gate, regulating the entry and exit of proteins and lipids. This compartmentalization is critical for maintaining the unique composition of the ciliary membrane, which differs significantly from the plasma membrane of the cell body.

The intraflagellar transport (IFT) system, consisting of molecular motors (kinesin-2 and dynein-2) and associated protein complexes (IFT-A and IFT-B), plays a pivotal role in assembling and maintaining the axoneme by ferrying protein cargo along the microtubules. The BBSome, a protein complex encoded by Bardet–Biedl syndrome genes, cooperates with the IFT system to regulate membrane protein trafficking within the cilium. Disruption of the IFT and BBS machinery leads to severe defects of the ciliary structure and dynamics, and is implicated in a variety of human diseases. Ciliary extracellular vesicles shed from the ciliary body contribute to intercellular communication and regulation of ciliary composition by selectively exporting membrane proteins and signaling molecules.

### Ciliogenesis and ciliary dynamics

Primary cilia are highly dynamic structures for which the formation, disassembly, and length are tightly regulated in response to both intra- and extracellular cues. In proliferating cells, cell cycle-dependent regulation plays a central role. Ciliogenesis predominantly occurs during the G0/G1 phase, whereas disassembly is typically triggered as cells re-enter the cell cycle, particularly during the G1/S transition or prior to mitosis (Gopalakrishnan et al., 2023).

Proper ciliogenesis requires targeted delivery of the membrane and protein components to the basal body. Vesicle trafficking is a key mechanism supporting this process, mediating cargo transport from the Golgi apparatus to the vicinity of the basal body (Wang and Dynlacht, 2018). This trafficking system is orchestrated by the Ras-related proteins in brain (Rab) family of small GTPases, which act as molecular switches in the coordination of vesicle budding, movement, and fusion. The activity of Rab proteins is further modulated by upstream regulators such as guanine nucleotide exchange factors (GEFs) and GTPase-activating proteins (GAPs).

The length and maintenance of primary cilia are also dynamically regulated by various physiological and pathological processes (Macarelli et al., 2023). Representative external factors that influence ciliary length include various cytokines, serum concentration in the culture medium, drugs, and oxygen levels. One of the key mechanisms regulating ciliary length involves an increase in intracellular calcium ion concentration, which activates Aurora A kinase (AurKA). AurKA subsequently activates histone deacetylase 6 (HDAC6), a tubulin deacetylase that destabilizes axonemal microtubules and promotes cilium shortening and resorption (Macarelli et al., 2023).

### Ciliary signaling pathways and core components

Primary cilia serve as highly specialized signaling hubs that coordinate numerous developmental and homeostatic processes by concentrating receptors and signaling mediators in a spatially compartmentalized manner (Anvarian et al., 2019). Several key signaling pathways require the cilium for proper activation and function. Understanding the molecular architecture is essential for interpreting later sections of this review, which describe how these pathways are altered in ALS.

One of the best-characterized cilia-dependent pathways is the Shh signaling pathway. In this pathway, the Shh ligand binds to the receptor Patched1 (PTCH1), releasing the inhibition of Smoothened (SMO), which then accumulates in the ciliary membrane. This leads to the activation and processing of glioma-associated oncogene homolog (GLI) transcription factors (GLI1, GLI2, GLI3). Shh signaling is essential not only for neural development and tissue patterning, but also for the maintenance of motor neurons in adult mammals (Peterson and Turnbull, 2012).

The Wnt signaling pathway is closely associated with both primary cilia and ALS. Wnt ligands, such as Wnt1, Wnt3a, and Wnt5a, bind to Frizzled (FZD) receptors and their co-receptors to activate downstream signaling. Canonical Wnt signaling leads to the stabilization of β-catenin, which translocates to the nucleus to regulate gene expression. In contrast, non-canonical pathways, including Wnt/planar cell polarity and Wnt/Ca^2+^ signaling, function independently of β-catenin and are typically driven by ligands like Wnt5a. The relationship between primary cilia and Wnt signaling remains controversial, and no definitive evidence has yet been obtained that primary cilia directly govern the Wnt pathway. Nevertheless, many studies have presented experimental observations indicating that Wnt signaling is modulated by primary cilia. Several key Wnt pathway components, such as Disheveled (DVL), FZD, inversin, and glycogen synthase kinase 3 (GSK3), are localized to the cilium, highlighting a close connection between primary cilia and Wnt signaling regulation.

The TGF-β signaling pathway also relies on ciliary localization for its regulation (Christensen et al., 2017). TGF-β ligands (TGF-β1, -β2, -β3) bind to TGF-β receptors (TGF-βR1 and TGF-βR2), which activate Sma and Mad related protein (SMAD) 2/3 transcription factors that subsequently complex with SMAD4. TGF-β receptors and phospho-SMADs have been found enriched at the ciliary base. This localization is essential for controlling signaling strength and specificity, particularly in processes such as autophagy, epithelial-to-mesenchymal transition, and central nervous system (CNS) inflammation.

Other signaling pathways are also coordinated by primary cilia (Ma et al., 2022). The Notch signaling pathway and platelet-derived growth factor (PDGF) signaling pathway are regulated by primary cilia. In addition, several cilia-localized G protein-coupled receptors (GPCRs), such as adenylyl cyclase type 3 (AC3), serotonin 5-HT6 receptor, and somatostatin receptor 3 (SSTR3), play roles in neuromodulation and are increasingly recognized as important mediators of ciliary signaling. Moreover, metabolic pathways such as the mechanistic target of rapamycin (mTOR) pathway are modulated and influenced via cilia-associated inputs, linking cilia to cellular metabolism and autophagy.

These signaling pathways highlight the essential role of primary cilia in integrating extracellular cues to coordinate intracellular responses. A foundational understanding of these signaling mechanisms is critical, as disruptions of these signaling pathways have been increasingly implicated in the pathogenesis of ALS.

### Signal reception, transduction, and modulation by primary cilia

The primary cilium serves as a critical sensory organelle that detects external cues and transduces them into intracellular signaling pathways (Mill et al., 2023; Nishimura et al., 2019). These cues include not only chemical signals such as growth factors and hormones, but also physical stimuli such as changes in temperature and fluid shear stress. In the nervous system, primary cilia are particularly involved in sensing ligands such as Hedgehog proteins and neuropeptides.

Alterations in the formation, disassembly, or length of primary cilia can profoundly influence the reception and modulation of these signals. For instance, the emergence or elongation of primary cilia has been associated with heightened responsiveness to Shh ligands (Bangs and Anderson, 2017). Conversely, in pathways such as Wnt and mTOR, the presence of primary cilia is often linked to a suppressive effect on signal activation. However, even within a single signaling pathway, the relationship between ciliary structure and signaling activity remains controversial, with inconsistent observations reported in the literature.

These discrepancies highlight the context-dependent nature of cilia-signaling interactions, which can vary depending on factors such as cell type, differentiation status, and environmental conditions. Moreover, technical limitations–particularly in assessing signaling dynamics *in vivo*–further complicate our understanding of primary cilia biology. Nevertheless, numerous studies suggests that primary cilia play an essential role in regulating diverse aspects of cellular metabolism and function, and achieve this by integrating external stimuli and modulating intracellular signal transduction. Importantly, abnormal ciliary formation, loss, or alterations in ciliary length can disrupt normal signaling homeostasis, contributing to the pathogenesis and progression of various diseases, including ALS.

### Roles of primary cilia in adult and aging brain

Primary cilia play multiple roles throughout the lifespan of the brain, from early neurodevelopment to the processes of aging. During embryonic and postnatal development, these cilia function as essential signaling hubs for pathways such as Shh, Wnt, and Notch, which orchestrate neural patterning, neuronal subtype specification, and the balance between proliferation and differentiation of neural progenitor cells.

The current understanding of the structures and functions of primary cilia in the mature nervous system remains limited (Sterpka and Chen, 2018). In the postnatal and adult brain, primary cilia are consistently observed on neurons and astrocytes. They are also present on oligodendrocyte precursor cells, whereas mature oligodendrocytes generally lack them. In addition, vascular endothelial cells harbor primary cilia, which may function as mechanosensors of blood flow. Primary cilia have also been identified on neural stem cells in the hippocampal dentate gyrus and the subventricular zone, where they regulate proliferation and differentiation. However, knowledge regarding potential differences in ciliary length, prevalence, or susceptibility across brain regions remains scarce.

Neuronal primary cilia typically show high expression of AC3, which has been implicated in the regulation of neuronal activity. Genetic variants and mutations in AC3 have been associated with conditions such as depression, autism spectrum disorders, and obesity in genetic association studies. In contrast, astrocytes remain dynamic even in adulthood, and primary cilia in these cells could contribute to the regulation of cell division and other proliferative processes such as astrogliosis, regeneration, and inflammation (Ki et al., 2021).

With aging, structural and functional alterations of primary cilia have been documented in certain brain regions, although the evidence remains scarce. For example, in mouse hippocampal neurons, aging has been associated with reduced expression of core ciliary proteins and, paradoxically, elongation of ciliary axonemes. These changes have been linked to decreased autophagy and impaired memory function (Rivagorda et al., 2025). By contrast, in the rat hypothalamus, primary cilia expressing the melanocortin-4 receptor, which is important for regulating feeding and metabolism, have been reported to shorten with age, a change proposed to contribute to age-related obesity (Oya et al., 2024). Collectively, such observations support the concept that age-related degeneration of primary cilia may contribute to late-onset diseases and functional decline, leading to the proposed notion of “age-related ciliopathy” (Oya et al., 2024).

In summary, primary cilia play essential roles in the brain and nervous system across the lifespan. However, many aspects of primary ciliary functions in adulthood and aging remain unresolved. Notably, while primary cilia have been identified on motor neurons (the primary site of pathology in ALS), their specific functional roles in this cell type are not understood and warrant further investigation. The investigations into ALS and cilia introduced below are considered important not only for elucidating the pathogenesis of ALS, but also for advancing the understanding of the relationships between the motor neuron system and primary cilia.

## Emerging genetic links between primary ciliogenesis and ALS

Until recently, knowledge regarding structural changes to primary cilia in ALS has remained limited, with only a few reports available from studies using SOD1 mice. However, recent insights have revealed that mutations in ALS-associated genes are significantly involved in ciliogenesis, leading to growing attention toward the relationship between primary cilia and ALS (Table 1). In this section, we first introduce the earliest study that reported alterations of primary cilia in mutant SOD1 mice. Next, we discuss the cilia-related roles of two ALS-associated genes, *NEK1* and *C21orf2* (Figure 1B). We also introduce emerging evidence on the relationship between primary cilia and the key ALS-associated gene *C9orf72*.

**TABLE 1:** Summary of ALS-associated genes linked to ciliary structure or function.

Gene	Ciliary localization site	Cilia-related function	Cilia-related phenotype in ALS	ALS-associated mutations	Experimental models used	References
SOD1	Not reported in ciliary context	Not reported	Ciliary shortening, impaired Shh signaling	G93A	Mutant SOD1 mice	Ma et al., 2011
NEK1	Basal body	Promotes ciliogenesis; regulates ciliary disassembly	Ciliary shortening due to increasing intracellular calcium with activation of AurkA-HDAC6 pathway, impaired Shh signaling	p.E853Rfs*9, p.M1?, p.Q132=	patient-derived fibroblasts, NSC-34, iPSC-derived motor neurons	Noh et al., 2025
C21orf2	Basal body	Interacts with NEK1; regulates ciliogenesis	Ciliary shortening, impaired Shh response, reduction of CRABP1 expression	p.V58L, p.Y68*, p.R106C, p.R172W, p.A255T	SH-SY5Y cells, iPSC-derived motor neurons	De Decker et al., 2025
C9orf72	Endosomes, vesicles	GAP for RAB8A; negatively regulates ciliogenesis	Ciliary elongation, enhanced Shh signaling response (in knock-down models); ciliary shortening (iPSC-MN and autopsy samples)	GGGGCC (G4C2) hexanucleotide repeat expansion in intron 1	HEK293T, ARPE-19, NIH3T3, iPSC-derived motor neurons, C9orf72-KO mice, autopsy brain sample	Tang et al., 2023; De Decker et al., 2025
hnRNPA1	Not reported in ciliary context	RNA splicing of ciliary genes	Inducing RNA splicing changes of genes associated with primary cilia	D262V	SH-SY5Y cells	Lee and Rio, 2024
TDP-43	Pericentriolar matrix	Local RNA regulator at centrosome; potential role in mRNA trafficking	Not reported	Not reported	Human skin fibroblasts, SH-SY5Y cells, HeLa, U-87 MG, KE-37	Bodin et al., 2023
p97/VCP	Pericentriolar region	Promotes ciliogenesis by removing CP110; regulates autophagy	Not reported	Not reported	hTERT-immortalized retinal pigment epithelial cells, MEF cells	Dewanji et al., 2025
p62/SQSTM1	Not reported in ciliary context	Suppresses ciliogenesis via DVL2–AurKA signaling; involved in autophagy regulation	Not reported	Not reported	NIH3T3 cells	Mun et al., 2020
DCTN1	Basal body	Required for dynein-mediated retrograde transport	Not reported	Not reported	hTERT-immortalized retinal pigment epithelial cells	Chen et al., 2015
TUBA4A	Axoneme	Component of axonemal microtubules	Not reported	Not reported	Trabecular meshwork cells	Shim et al., 2021

This table summarizes how key pathological processes in ALS (including proteostasis failure, RNA metabolism dysfunction, microtubule instability, neuroinflammation, and mitochondrial impairment) interact with primary cilia. For each process, both directions of influence are described: how the ALS mechanism affects ciliary structure or function, and how ciliary dysfunction may in turn exacerbate the respective pathology. This overview highlights a potential vicious cycle linking primary cilia abnormalities and ALS progression. ARPE-19, human retinal pigment epithelial cell line; AurKA, Aurora kinase A; C21orf2, chromosome 21 open reading frame 2; C9orf72, chromosome 9 open reading frame 72; CP110, centriolar coiled-coil protein of 110 kDa; DCTN1, dynactin subunit 1; DVL2, Disheveled segment polarity protein 2; GAP, GTPase-activating protein; hTERT, human telomerase reverse transcriptase; HDAC6, histone deacetylase 6; hnRNPA1, heterogeneous nuclear ribonucleoprotein A1; iPSC, induced pluripotent stem cell; KE-37, human T lymphoblast cell line; NEK1, NIMA-related kinase 1; NSC-34, hybrid motor neuron-like cell line; RAB8A, Ras-related protein Rab-8A; SH-SY5Y, human neuroblastoma cell line; SMCR8, Smith–Magenis syndrome chromosome region candidate 8; SQSTM1, sequestosome 1; TUBA4A, tubulin alpha-4A; U-87 MG, human glioblastoma cell line; VCP, valosin-containing protein.

### SOD1

*Superoxide dismutase 1* was the first gene identified with a relationship to ALS, and G93A SOD1 mutant mice remain the most widely used model for the disease. Ma et al. (2011) hypothesized and examined the involvement of primary cilia in the pathogenesis of ALS using mutant SOD1 mice. When spinal motor neurons derived from embryonic mice were cultured, the proportion of cells showing primary cilia was significantly reduced compared to controls. *In vivo* analysis also confirmed a reduction in the number of primary cilia on spinal motor neurons, suggesting a potential contribution of ciliary abnormalities to the pathogenesis of ALS.

An issue of note, however, is that these SOD1 mutant mice lacks TDP-43 inclusions, a hallmark seen in most ALS patients. Care should therefore be taken in generalizing evidence from SOD1 mice to the broader ALS pathology. Moreover, no studies have yet demonstrated that SOD1 directly regulates ciliogenesis. Nonetheless, insights obtained from SOD1 mice have significantly advanced our understanding of ALS and serve as the basis for the current focus on mutations in cilia-related genes.

### NEK1

*NIMA-related kinase 1* was identified as an ALS-related gene through whole-exome sequencing (Kenna et al., 2016). A meta-analysis revealed *NEK1* mutations in 3.1% of ALS patients, with an odds ratio of 2.14 (Yao et al., 2021). While clinical features specific to NEK1-ALS have rarely been reported, some cases with a frail-arm phenotype or accompanying behavioral impairments have been described. In neuropathological studies, phosphorylated TDP-43-positive inclusions were detected in ALS cases showing *NEK1* mutations.

NIMA-related kinase 1 has been implicated in primary ciliogenesis, cell cycle regulation, microtubule homeostasis, DNA repair, and nuclear transport (Watanabe et al., 2025). The role of this protein in primary ciliogenesis was originally noted in studies of polycystic kidney disease. Although the precise mechanisms by which NEK1 is involved in ciliogenesis remain unclear, it is typically enriched at the basal body and forms a complex with C21ORF2, suggesting that this localization is important (Watanabe et al., 2025). Homozygous *NEK1* mutations can cause severe skeletal dysplasia (short-rib thoracic dysplasia type II), which shares features with other ciliopathies. In ALS, most mutations are heterozygous and include both loss-of-function and missense variants.

A recent study reported that three ALS-associated *NEK1* mutations caused structural abnormalities in primary cilia, impaired Shh signaling, and promoted cell cycle reentry (Noh et al., 2025). In addition, mutant cells exhibited enhanced calcium influx and activation of the Aurora A–HDAC6 pathway, resulting in disassembly of the cilium. These observations suggest that *NEK1* mutations may contribute to ALS pathogenesis through ciliary dysfunction in addition to other pathogenic mechanisms.

### C21orf2

Chromosome 21 open reading frame 2, also known as cilia and flagellar associated protein 410 (CFAP410), was identified as an ALS-associated gene around the same time as NEK1 (van Rheenen et al., 2016). Burden analysis in genome-wide association study (GWAS) studies showed that heterozygous mutations in C21orf2 were found in 2%–3% of ALS patients. Homozygous mutations in C21orf2 are known to cause retinal dystrophy and have also been linked to congenital syndromes involving polydactyly and skeletal dysplasia, all of which are typical features of ciliopathies. A unique case report described sibling patients with retinal dystrophy and subsequent development of ALS who carried a homozygous C21orf2 mutation (Kurashige et al., 2020).

Chromosome 21 open reading frame 2 functions as a binding partner of NEK1 and is involved in primary ciliogenesis, DNA damage repair, mitochondrial metabolism, and neuronal excitability (Watanabe et al., 2025; Zelina et al., 2024). As with NEK1, C21ORF2 localizes to the basal body of primary cilia. ALS-associated mutations in C21ORF2 have been reported to both promote NEK1 degradation and enhance its stability, resulting in accumulation (Watanabe et al., 2025; Zelina et al., 2024). This implies that the interaction between NEK1 and C21ORF2 might be altered depending on cell type and conditions.

A recent study demonstrated that knockdown of *C21orf2* in SH-SY5Y and induced pluripotent stem cell (iPSC)-derived motor neurons reduced both the frequency and length of primary cilia (De Decker et al., 2025). This phenotype is rescued by re-expression of wild-type *C21orf2*, but not by ALS-associated mutant forms, indicating a failure of the mutant proteins to support normal ciliogenesis. These results suggest that *C21orf2* mutations impair the structure and function of primary cilia, contributing to ALS pathogenesis.

### C9orf72

*Chromosome 9 open reading frame 72* is the most common genetic cause of familial ALS, identified in 2011. In European cohorts, C9orf72 repeat expansions account for nearly half of familial ALS cases and about 5%–10% of sporadic cases, whereas in Asian populations they are comparatively rare, reflecting differences in genetic background across populations. Hexanucleotide (GGGGCC) repeat expansions in this gene cause various molecular disturbances. Transcriptional interference due to the repeat structures reduces C9ORF72 expression, leading to a loss-of-function effect. At the same time, the repeat-containing RNA forms RNA foci that sequester RNA-binding proteins, disrupting nuclear metabolism and protein synthesis. In addition, repeat-associated non-AUG translation generates dipeptide repeat proteins, which exert toxic gain-of-function effects.

Since its discovery, C9ORF72 has been shown to have physiological functions, particularly in regulating vesicle trafficking. It forms a complex with Smith–Magenis syndrome chromosome region, candidate 8 (SMCR8) and WD repeat-containing protein 41 (WDR41), acting as a modulator of RAB GTPases involved in trafficking. Among these, RAB8 is a critical factor in promoting ciliogenesis.

A recent report indicated that the C9orf72-SMCR8 complex functions as a GAP, negatively regulating RAB8A activity and thereby suppressing ciliogenesis (Tang et al., 2023). Knockout of either C9ORF72 or SMCR8 leads to increases in both cilia frequency and length. These observations suggest that loss of C9ORF72 disrupts ciliary control, potentially contributing to pathological mechanisms in C9orf72-related ALS. Notably, elongated and more frequent primary cilia were observed not only in the brain but also in renal and splenic cells, indicating that ciliary abnormalities extend to peripheral organs. These findings raise the possibility that C9orf72-linked diseases might be conceptualized as multisystem ciliopathies rather than disorders confined to the central nervous system.

However, motor neurons derived from C9orf72-ALS patient iPSCs have shown no reduction in C9ORF72 protein levels and instead exhibit shortened primary cilia (De Decker et al., 2025). A similar reduction in ciliary length was also observed in the motor cortex of autopsy samples from C9orf72-ALS patients (De Decker et al., 2025). This raises the possibility of a gain-of-function effect on cilia. Although an increasing number of studies report ciliary changes associated with C9orf72, the results have not been entirely consistent, and further investigation is needed to elucidate the underlying mechanisms.

## Dysregulation of ciliary signaling in ALS: TGF-β, Wnt, and shh signaling

Primary cilia dysfunction could influence multiple cellular signaling pathways in the neural system. This section focuses on Shh signaling, TGF-β signaling, and Wnt signaling, which are closely related to primary cilia and have been frequently implicated in ALS. Interventions targeting these signaling pathways are attracting attention as promising therapeutic strategies for ALS, and restoring or modulating primary cilia may represent an effective approach.

### Shh signaling

Sonic Hedgehog signaling is the pathway most intimately associated with primary cilia. The abnormalities in ciliogenesis caused by mutations or knockdown of *NEK1, C21orf2*, or *C9orf72* described earlier are also accompanied by alterations in Shh signaling (Tang et al., 2023; De Decker et al., 2025; Noh et al., 2025). Loss of C9ORF72 leads to an increased frequency and elongation of primary cilia, thereby enhancing cellular responsiveness to Shh stimulation (Tang et al., 2023). In contrast, defects in NEK1 or C21ORF2 impair primary ciliogenesis, resulting in attenuated Shh signaling (De Decker et al., 2025; Noh et al., 2025). Furthermore, mutations in *C21orf2* or *C9orf2* reduce the expression of cellular retinoic acid binding protein 1 (CRABP1), a key Shh-regulated gene essential for motor neuron function, survival, and neuromuscular junction (NMJ) formation (De Decker et al., 2025). Indeed, motor neurons derived from patients with *C21orf2* mutations exhibit impaired NMJ formation and reduced survival. These observations suggest that cilia-related defects in ALS are directly linked to Shh signaling alterations and may contribute to motor neuron degeneration and cell death.

In addition, several studies have shown that Shh signaling directly supports motor neuron survival. For example, Shh promoted motor neuron survival, neurite outgrowth, and ciliogenesis in primary cultures derived from both G93A SOD1 and wild-type mice (Ma et al., 2013). Furthermore, Shh and its agonists were seen to enhance motor neuron survival under conditions of oxidative stress induced by hydrogen peroxide (Peterson and Turnbull, 2012). Although evidence remains limited, these observations suggest that activation of Shh signaling may represent a promising therapeutic avenue in ALS.

### TGF-β Signaling

Transforming growth factor-β signaling is increasingly recognized as a key player in ALS pathogenesis, showing elevated expression in serum, spinal cord, and muscle of ALS patients and models. Importantly, TGF-β exhibits a dual, context-dependent role, exerting both neuroprotective and neurotoxic effects.

On the protective side, Smad2/3 activation and nuclear translocation have been observed in the motor neurons of ALS patients and SOD1 mutant mice (Nakamura et al., 2008, 2013). Overexpression of Smad2 reduced the amount of TDP-43 cytoplasmic aggregates in HEK293T cells (Nakamura et al., 2013). TGF-β/Smad signaling supports protein homeostasis by enhancing autophagy and suppressing the aggregation of misfolded proteins such as mutant SOD1 and TDP-43 (Zhang et al., 2020). Moreover, in C9orf72-linked ALS, TGF-β1 expression in patient-derived motor neurons mitigates glutamate-induced toxicity (Milioto et al., 2024).

Conversely, astrocyte-derived TGF-β1 can aggravate disease by promoting neuroinflammation. In SOD1 mutant mice, pharmacological inhibition of TGF-β signaling slows disease progression (Endo et al., 2015). In microglia, TGF-β signaling is also activated, accompanied by upregulation of CD109, a negative regulator of the pathway (Li et al., 2025). Targeting CD109 partially alleviates neuroinflammation. Similarly, TGF-β knockdown improves neurodegeneration in zebrafish models of ALS (Gonzalez et al., 2024).

Although direct evidence linking primary cilia to TGF-β signaling in ALS remains scarce, these studies suggest that ciliary dysfunction in ALS may modulate TGF-β signaling dynamics, representing an underexplored mechanism warranting further investigation.

### Wnt Signaling

Wingless/Integrated (Wnt) is considered as a cilia-associated signaling pathway. In both ALS models and patient tissues, the expression patterns of Wnt ligands and receptors are frequently altered (Gonzalez-Fernandez et al., 2020).

Many studies have reported activation of the canonical Wnt/β-catenin pathway in ALS, which has been implicated in neuronal degeneration and glial cell proliferation (Jiang et al., 2021). Astrocyte-derived Wnt5a is often upregulated in ALS and contributes to neuroinflammation by activating microglial cells (Gonzalez-Fernandez et al., 2020). On the other hand, downregulation of the Wnt1/β-catenin signaling pathway was associated with reduced neuronal viability in mutant SOD1 model cells (Pinto et al., 2019). This attenuation of signaling was linked to the abnormal cytoplasmic accumulation and mislocalization of β-catenin, potentially leading to impaired cell–cell interactions. Taken together, these observations suggest that Wnt signaling, like TGF-β signaling, plays a dual role in ALS pathophysiology, acting both as a neuroprotective mechanism and as a contributor to disease progression.

Emerging evidence also links dysregulated Wnt signaling to TDP-43 mislocalization, a hallmark of ALS, via disrupted nucleocytoplasmic transport (Zhang et al., 2024). In addition, ectopic P-granules 5 (EPG5) deficiency, which impairs endosomal trafficking and alters Wnt signaling, induced motor neuron degeneration in mice, suggesting interplay between Wnt, vesicle dynamics, and ALS pathogenesis (Yuan et al., 2025).

Although direct evidence linking Wnt signaling alterations to primary cilia degeneration in ALS is currently lacking, the strong ciliary association of Wnt signaling in other systems, together with its dysregulation in ALS, highlights this pathway as a promising target for future cilia-focused investigations.

In summary, the major ciliary signaling pathways implicated in ALS include Shh, TGF-β, and Wnt, each showing complex roles in motor neuron survival and disease progression. An important point is that these ciliary signaling pathways are not independent but exhibit substantial cross-talk. For instance, Shh, Wnt, Notch, and TGF-β signaling converge at the primary cilium and influence each other in regulating neural development and homeostasis. Thus, disruption of ciliogenesis in ALS may simultaneously disturb multiple pathways, amplifying pathogenic effects.

## Interplays between primary cilia dysfunctions and ALS pathogenesis

**FIGURE 2:**
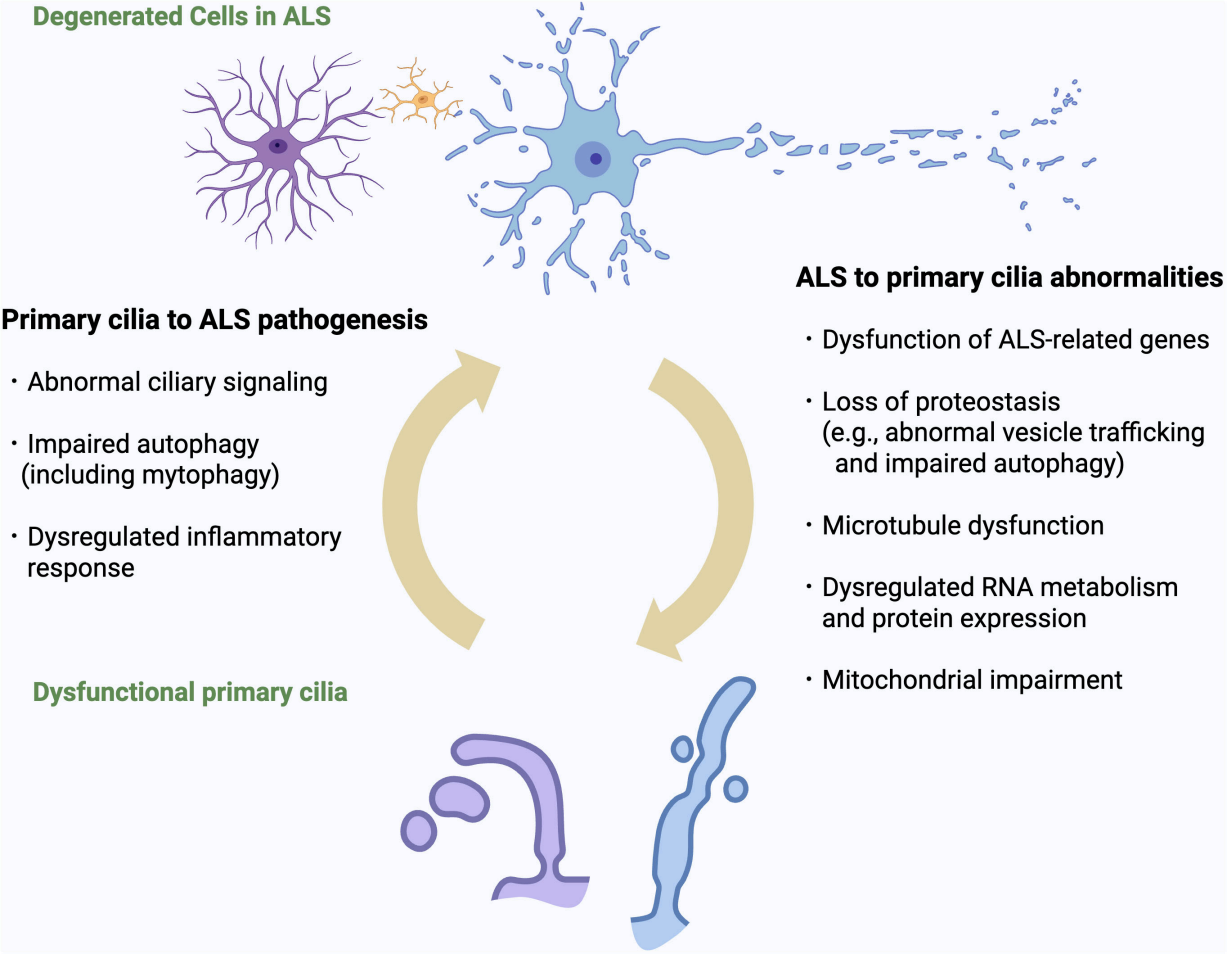
Bidirectional relationship between primary cilia dysfunction and ALS pathomechanisms. Overview of the bidirectional interactions between primary cilia dysfunction and ALS-related cellular processes. ALS-related mechanisms (including dysfunction of ALS genes, impaired proteostasis, RNA dysregulation, microtubule instability, neuroinflammation, and mitochondrial impairment) can induce structural and functional ciliary abnormalities. Conversely, defective cilia can exacerbate ALS pathogenesis by impairing ciliary signaling, autophagy and inflammatory regulation. This vicious cycle may represent a novel pathogenic cascade in ALS.

In this section, we focus on abnormal RNA metabolism, loss of proteostasis, microtubule dysfunctions, neuroinflammation, and mitochondrial dysfunction, all of which are known to be associated with both ALS and cilia (Hu et al., 2024; Table 2). Importantly, many of these pathological processes can either induce ciliary defects or are themselves triggered by ciliary dysfunction (Figure 2). This bidirectional or potentially cyclical relationship between ALS and cilia may be a key element in advancing our understanding of disease mechanisms and identifying novel therapeutic targets.

**TABLE 2:** Bidirectional interactions between ALS-related pathological mechanisms and primary cilia.

ALS-related pathomechanism	Impact on primary cilia	Feedback from ciliary dysfunction	Key references
Loss of proteostasis	Impaired ciliogenesis due to disrupted vesicle trafficking, aberrant autophagy, and ALS mutated genes (e.g., VCP and p62/SQSTM1)	Impaired autophagy and lysosomal function	Dewanji et al., 2025; Mun et al., 2020
Abnormal RNA metabolism	Aberrant cilia-related gene expression due to dysfunctional splicing factors	Not well understood	Wheway et al., 2015; Bodin et al., 2023; Lee and Rio, 2024
Microtubule dysfunction	Influence on cilium structure due to cytosolic microtubule instability and concentration change	Not well understood	Sharma et al., 2011; Chen et al., 2015
Neuroinflammation	Alteration of cilium length due to lipopolysaccharide and pro-inflammatory cytokines	Dysregulated inflammatory response via ciliary length change and ciliary signaling	Baek et al., 2017; Muhamad et al., 2024; Guttenplan et al., 2020
Mitochondrial dysfunction	Defective intraflagellar transport and dysregulation of ciliary gene expression	Impaired mitophagy and metabolic signaling	Ignatenko et al., 2023; Bae et al., 2019; Silva and Cavadas, 2023

List of ALS-related genes with experimentally supported roles in ciliogenesis, ciliary signaling, or centrosomal function. The table includes the known function and localization in primary cilia (e.g., basal body, centrosome), and evidence for ciliary involvement from ALS models and cellular experiments. This summary supports the hypothesis that ciliary dysregulation may be a common mechanism underlying multiple ALS genotypes. VCP, valosin-containing protein; SQSTM1, sequestosome 1.

### Loss of proteostasis

Disruption of proteostasis is a major pathological mechanism in ALS. Misfolded proteins such as TDP-43, FUS, and SOD1 accumulate and form cytoplasmic inclusions due to impaired clearance by the ubiquitin-proteasome system (UPS) and autophagy. Several genes linked to ALS are directly involved in proteostasis, such as valosin-containing protein (VCP), sequestosome 1 (SQSTM1), ubiquilin 2 (UBQLN2), and optineurin (OPTN).

Recent studies have suggested that several ALS-causative genes involved in the regulation of proteostasis also play roles in the formation and disassembly of primary cilia. The p97/VCP is essential for initiating ciliogenesis by removing centriolar coiled-coil protein of 110 kDa (CP110), a capping protein that inhibits axoneme elongation, from the mother centriole (Dewanji et al., 2025). In addition, p62/SQSTM1 has been shown to suppress ciliogenesis via activation of the DVL2–AurKA signaling pathway (Mun et al., 2020). These observations suggest that the proteostasis dysfunction and these gene mutations observed in ALS may also contribute to abnormalities in primary cilia.

Among the protein clearance systems reported to be impaired in ALS, autophagy is particularly closely associated with primary cilia, and the two are reciprocally related. Under basal conditions, autophagy suppresses ciliogenesis by degrading IFT20. In contrast, under conditions such as nutrient starvation, inhibitory factors of primary ciliogenesis, including oral-facial-digital Syndrome 1 protein (OFD1) and myosin heavy chain 9, non-muscle (MYH9), are selectively degraded by activated autophagy, thereby promoting ciliogenesis (Tang et al., 2013). Thus, primary cilia are influenced by autophagy in a context-dependent manner.

Conversely, autophagy can be induced by primary cilia. Shh signaling has been shown to activate autophagy. When primary ciliogenesis is impaired, this signaling cannot be properly activated, resulting in reduced autophagic activity. Similarly, defective primary ciliogenesis can lead to excessive activation of mTOR signaling, which also exerts an inhibitory effect on autophagy. Therefore, considering the reciprocal interactions between primary cilia and autophagy, a precise understanding of their relationship is of particular importance in elucidating the pathogenesis of ALS.

Taken together, these reciprocal influences suggest that autophagy–cilia crosstalk is not only mechanistically relevant but may also represent a key amplifier of proteostasis failure in ALS. Collectively, these observations support the existence of a vicious cycle in which failure of proteostasis disrupts the structure and function of primary cilia, while ciliary dysfunction further impairs protein clearance mechanisms, ultimately accelerating neurodegeneration in ALS.

### Abnormal RNA metabolism

Dysregulation of RNA metabolism is a central pathogenic mechanism in ALS. RNA-binding proteins (RBPs) such as TDP-43, FUS, heterogeneous nuclear ribonucleoprotein A1 (hnRNPA1), and T-cell intracellular antigen-1 (TIA1) are key regulators of splicing, mRNA transport, stability, and localized translation. In ALS, these RBPs are often mislocalized to the cytoplasm and aggregate, leading to widespread disruption of RNA processing and stress granule dynamics. TDP-43 pathology, present in most cases of ALS, underscores the critical role of RNA metabolism dysfunction in motor neuron degeneration.

Aberrant RNA splicing has been increasingly implicated in impairing ciliogenesis (Wheway et al., 2015). Several splicing factors–such as pre-mRNA-processing-splicing factor (PRPF)8, PRPF31, and SON–have been shown to be essential for primary cilia formation, and mutations in these genes cause ciliopathies such as retinitis pigmentosa and intellectual disability (Wheway et al., 2015). Given that ALS-associated RBPs regulate splicing, splicing abnormalities in ALS could plausibly disrupt ciliogenesis indirectly by altering the expression or processing of key ciliary genes. Indeed, a very recent study demonstrated that the ALS-associated hnRNPA1 D262V mutation induced widespread splicing changes, many of which involve genes related to ciliogenesis, further supporting the potential link between ALS pathogenesis and primary cilia (Lee and Rio, 2024).

Importantly, a recent study has identified TDP-43 enrichment at the pericentriolar matrix, the sub-centrosomal region that anchors the basal body during ciliogenesis (Bodin et al., 2023). TDP-43 colocalizes at the centrosome with centrosomal RNAs and proteins, including ALS-associated molecules such as dynactin subunit 1 (DCTN1), VCP, and ataxin 2 (ATXN2). This observation suggests that TDP-43 may regulate localized translation or mRNA transport near the ciliary base. TDP-43 dysfunction at this site could impair the local proteostasis or mRNA dynamics required for ciliogenesis and proper functions of motor neurons, offering a potential mechanistic link between ALS-related defects in RNA metabolism and primary cilia dysfunction.

Collectively, these observations support the hypothesis that abnormal RNA metabolism contributes to ciliary defects in ALS, either via splicing errors or through disrupted local RNA regulation at the centrosome.

### Microtubule dysfunction

In ALS, cytoskeletal abnormalities, particularly those related to microtubules, play an important role in disease development and progression. Impaired microtubule stability disrupts axonal transport, leading to progressive degeneration of distal motor neuron processes.

Several ALS-associated genes are involved in microtubule formation and function. For example, mutations in *Tubulin Alpha 4a (TUBA4A)*, which encodes α-tubulin, could induce microtubule disintegration. Mutations in *Kinesin Family Member 5A (KIF5A)*, which encodes a kinesin motor protein responsible for anterograde transport, were detected in ALS patients. *DCTN1* is essential for dynein-mediated retrograde transport, and its deficiency exacerbates TDP-43 aggregation. Moreover, TDP-43 pathology is thought to contribute to cytoskeletal dysfunction by repressing the expression of *stathmin 2 (STMN2)*, a gene involved in microtubule repair. These alterations converge on a common mechanism of impaired axonal transport and microtubule instability, ultimately promoting motor neuron degeneration in ALS.

Primary cilia are microtubule-based structures, and their formation and function are highly dependent on cytosolic microtubule concentration and stability (Sharma et al., 2011). As previously noted, NEK1 and C21ORF2 are involved in both microtubule regulation and ciliogenesis (Watanabe et al., 2025). These observations strongly suggest that the microtubule dysfunction observed in ALS may affect primary cilia. However, while many microtubule-related ALS genes contribute to disease pathology primarily through the disruption of axonal transport in motor neurons, the IFT machinery, which is essential for ciliogenesis, operates via distinct proteins and mechanisms. For example, axonal transport predominantly employs kinesin-1 (e.g., the KIF5 family) and cytoplasmic dynein-1, whereas intraflagellar transport utilizes kinesin-2 (e.g., the heterotrimeric KIF3 complex) and cytoplasmic dynein-2 (also known as IFT dynein). Therefore, whether the microtubule-related defects observed in ALS lead to primary cilia abnormalities remains an open question that merits further investigation.

In hTERT-immortalized retinal pigment epithelial cells, DCTN1 is distributed at the basal body, and its depletion impaired ciliogenesis (Chen et al., 2015). TUBA4A has been reported as a component of the primary cilium axoneme (Shim et al., 2021). These observations suggest that it is worth exploring whether the ALS-associated mutations of these genes influence primary cilia in neural cells, which may provide further insights into the disease mechanisms.

### Neuroinflammation

Neuroinflammation is increasingly being recognized as a key contributor to the pathogenesis of ALS. Although motor neuron degeneration is the primary pathology in ALS, accumulating evidence indicates that non-neuronal cells (particularly microglia and astrocytes) play critical roles in disease progression. Glial cells expressing G93A SOD1 mutation show pro-inflammatory profiles, indicating active glial involvement in ALS via neuroinflammation. Elevated levels of pro-inflammatory cytokines such as tumor necrosis factor-alpha (TNF-α) and interleukin-6 (IL-6) are frequently observed in serum from ALS patients, and activated microglia and astrocytes are frequently observed surrounding degenerating motor neurons in post-mortem tissues from ALS patients.

Neuroinflammatory responses can exacerbate excitotoxicity, mitochondrial dysfunction, and oxidative stress, all of which are implicated in ALS pathology. While inflammation may initially represent a protective response, chronic activation of immune pathways ultimately contributes to motor neuron death. Understanding the regulation of neuroinflammation and its cellular mediators is therefore essential for developing disease-modifying therapies in ALS.

Primary cilia play a role as critical coordinators of neuroinflammation. As previously described, ciliary signaling pathways such as TGF-β signaling and Wnt signaling are important regulators of neuroinflammation (Endo et al., 2015; Gonzalez-Fernandez et al., 2020). Lipopolysaccharide-induced inflammation in hippocampal neurons leads to primary ciliary shortening and activation of nuclear factor-kappa B (NF-κB) signaling (Baek et al., 2017). Silencing KIF3A, a motor protein essential for ciliogenesis, attenuates the lipopolysaccharide (LPS)-induced activation of NF-κB and downstream inflammatory responses. This result suggests that primary cilia serve as key modulators of CNS inflammation. A recent study showed that pro-inflammatory C3 astrocytes can induce inflammatory effects through the elongation of primary cilia (Muhamad et al., 2024). Given that C3 astrocytes are increased in ALS, and that knockdown of C3 astrocyte-specific genes delays disease progression in SOD1 mouse models, regulation of ciliary length might contribute to the modulation of neuroinflammation in ALS (Guttenplan et al., 2020). Taken together, these observations indicate that structural changes or dysfunction of primary cilia may influence the inflammatory environment in the ALS brain. Further investigation into how ciliary dynamics intersect with neuroinflammation could provide new insights into the mechanisms of ALS pathogenesis.

### Mitochondrial dysfunction

Mitochondrial dysfunction is a well-established pathological feature of ALS. Both animal and cell models have demonstrated abnormal mitochondrial morphology, impaired electron transport chain activity, and disrupted calcium-buffering capacity in motor neurons. Mutations in ALS-associated genes such as *SOD1, TARDBP, FUS, C9orf72*, and *Coiled-Coil-Helix-Coiled-Coil-Helix Domain Containing 10 (CHCHD10)* have been linked to disruption of the mitochondrial architecture, defects in axonal transport, and abnormal mitochondrial DNA transcription. Moreover, excess reactive oxygen species (ROS) derived from dysfunctional mitochondria contribute to protein misfolding and exacerbate neuronal injury. Imbalances in mitochondrial fission and fusion disrupt energy production and synaptic function, while impaired mitophagy leads to the accumulation of damaged mitochondria and accelerates neurodegeneration. These observations suggest that mitochondrial dysfunction is not merely a secondary consequence, but a driver of disease progression in ALS.

Recent evidence indicates that mitochondrial impairment may also affect the formation and maintenance of primary cilia. For example, mitochondrial depolarization or excess ROS can reduce both the length and frequency of primary cilia (Silva and Cavadas, 2023). Although the precise mechanisms remain unclear, some studies have proposed that insufficient ATP production may hinder the energy-dependent motor proteins required for IFT, thereby impairing ciliogenesis. A recent study showed that declines in mitochondrial oxidative phosphorylation in astrocytes activate transcription factors such as forkhead box J1 (FOXJ1) and regulatory factor X (RFX), both of which are known to promote motile ciliogenesis, leading to abnormal primary cilia elongation and distortion (Ignatenko et al., 2023).

Conversely, functional primary cilia actively regulate autophagy, including the selective removal of damaged mitochondria via mitophagy. Primary cilia dysfunction could disrupt this autophagy influx and has been implicated in other neurodegenerative diseases such as Parkinson’s disease, where impaired mitophagy contributes to disease pathology (Bae et al., 2019). Further, several cilia-mediated signaling pathways (including Shh, Wnt, and PDGF) are known to regulate mitochondrial metabolism. Disruption of these ciliary signaling pathways may therefore impair mitochondrial function.

Collectively, these observations support a bidirectional relationship: mitochondrial dysfunction may contribute to ciliary abnormalities, while ciliary dysfunction may in turn exacerbate mitochondrial damage. This cilia–mitochondria feedback loop could represent a critical mediator of neurodegeneration in ALS.

Taken together, these lines of evidence support the concept of a bidirectional relationship in which primary cilia dysfunction and ALS pathomechanisms mutually reinforce one another. As illustrated in Figure 2, proteostasis failure, RNA dysregulation, microtubule instability, neuroinflammation, and mitochondrial impairment can each induce ciliary abnormalities, while defective cilia in turn exacerbate these pathogenic processes. This vicious cycle provides a unifying framework for understanding how diverse ALS-associated mechanisms may converge on ciliary dysfunction, and highlights the need for further studies to determine whether breaking this cycle could represent a viable therapeutic strategy.

## Cilia-related therapeutics and clinical applications in ALS

Given the emerging role of primary cilia in regulating diverse cellular pathways implicated in neurodegeneration, there is growing interest in developing cilia-targeted strategies for therapeutic intervention and disease monitoring in ALS. In this section, we provide an overview of current approaches that consider primary cilia as clinical targets in ALS, which can be broadly categorized into three areas: modulation of ciliary signaling, regulation of ciliary structure and stability, and gene-based therapies.

### Ciliary signaling control

In ALS models, pharmacological activation of Sonic hedgehog (Shh) signaling enhances motor neuron survival, promotes ciliogenesis, and reduces apoptosis (Ma et al., 2013; Peterson and Turnbull, 2012). Accordingly, Shh ligands and agonists such as purmorphamine may represent potential therapeutic tools. Modulation of other ciliary pathways, including Wnt and TGF-β signaling, has also been reported to suppress neuroinflammation in ALS models (Endo et al., 2015; Nakamura et al., 2013). Although these pathways exhibit pleiotropic functions and require careful targeting, such findings demonstrate that pharmacological interventions acting on ciliary signaling are already available at the experimental level.

### Regulation of ciliary structure and stability

Strategies aimed at stabilizing or restoring cilia are also being explored. One of the most actively studied targets is HDAC6, a cytoplasmic enzyme that promotes ciliary disassembly by deacetylating α-tubulin. HDAC6 inhibitors such as tubastatin A have been shown to preserve ciliary length and function in various models (Noh et al., 2025). Notably, in ALS models, HDAC6 inhibition not only promotes ciliogenesis but also improves axonal transport and protein homeostasis, two core pathological processes of ALS (Guo et al., 2017). These dual effects highlight HDAC6 as a particularly promising therapeutic target. Additional approaches include modulation of mTOR signaling, where agents such as rapamycin and everolimus have been shown to promote ciliogenesis.

### Gene-based therapies

In diseases caused by ciliary gene defects, gene therapy offers a rational approach. AAV-mediated gene replacement has already been approved in retinal ciliopathies, demonstrating clinical feasibility. Antisense oligonucleotides (ASOs) represent another promising strategy; for example, ASO-based therapy is currently being tested in human trials for C9orf72-related ALS (McEachin et al., 2025), and similar approaches could potentially be extended to other ciliary ALS genes such as NEK1 or C21ORF2. The principal challenges for these therapies remain efficient delivery to the CNS and the minimization of off-target effects.

Overall, while therapeutic strategies directly targeting primary cilia are still limited in clinical practice, intensive research efforts are ongoing, and their potential relevance to ALS is expected to grow.

In parallel with therapeutic development, increasing attention has been directed toward the identification of reliable biomarkers for ALS. At present, the clinical diagnosis of ALS relies on neurological examination, electrophysiological testing, and the exclusion of alternative conditions. To complement these approaches and facilitate earlier diagnosis, quantitative biomarkers are increasingly required. Currently, the most established biomarkers in ALS are neurofilaments (neurofilament light chain and phosphorylated neurofilament heavy chain), which reflect axonal injury and correlate with diagnosis, prognosis, and treatment response.

By contrast, no clinical methods are available to directly assess primary cilia function in humans, including patients with ALS. Recently, ciliary extracellular vesicles, which are selectively secreted from primary cilia, have attracted attention as potential biomarkers (Vinay and Belleannée, 2022). These vesicles carry cilia-specific molecules such as ADP-ribosylation factor-like protein 13B (ARL13B) and cytochrome P450 oxidoreductase (POR) and may be detectable in cerebrospinal fluid or blood. If successfully implemented, this approach could not only provide insights into the contribution of ciliary dysfunction to disease progression, but might ultimately support earlier diagnosis in ALS.

## Future directions for investigating primary cilia in ALS

Finally, in this section we discuss several key areas for future research into the role of primary cilia in ALS. First, the relationship between primary cilia and ALS-related pathologies requires further exploration. Although several ALS-related genes have been implicated in ciliary dysfunction, including *NEK1*, *C21orf2*, and *C9orf72*, whether impaired ciliogenesis is a common feature across ALS subtypes remains unclear (Tang et al., 2023; De Decker et al., 2025; Noh et al., 2025). Further studies are warranted to examine how ALS-related mutations affect ciliogenesis and contribute to disease mechanisms. Determining whether ciliary degeneration represents a primary pathogenic event in ALS or arises secondarily in response to other cellular disturbances is also important, as this distinction has major implications for therapeutic targeting. No studies have investigated the causal relationship between the formation of TDP-43-positive inclusions, which are observed in the majority of ALS cases, and primary cilia dysfunction. Clarifying this point might be important for determining whether primary cilia abnormalities represent a common pathogenic mechanism in ALS.

Second, understanding the status of primary cilia in human ALS tissues, particularly in the sporadic cases that comprise the majority of patients, is a critical but underexplored area. Neuropathological studies using postmortem brain tissue are urgently needed. However, evaluating cilia in human tissues is technically challenging due to the microtubule-based nature of cilia, which makes them highly susceptible to ischemic artifacts and fixation delays in formalin-fixed samples. Protocols for reliable and reproducible cilia analysis in human brain tissue must therefore be developed and standardized. In addition, transcriptomic and proteomic profiling of human tissue samples and patient-derived iPSC models should be more broadly implemented.

**FIGURE 3:**
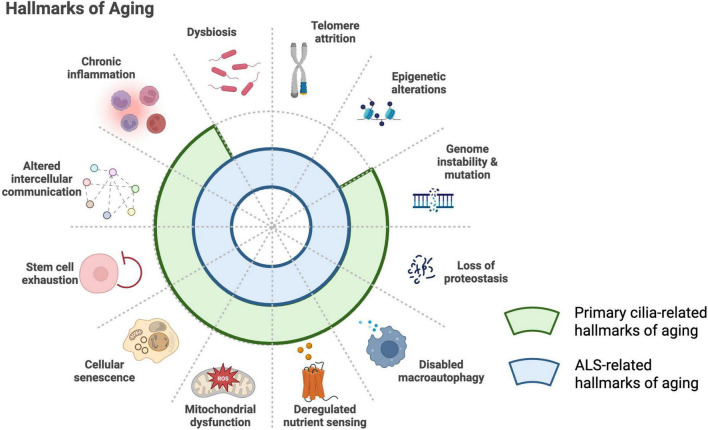
Shared hallmarks of aging in primary cilia biology and ALS. The established hallmarks of aging include loss of proteostasis, genomic instability, telomere attrition, epigenetic alterations, impaired macroautophagy, stem cell exhaustion, chronic inflammation, altered intercellular communication, dysbiosis, cellular senescence, deregulated nutrient sensing, and mitochondrial dysfunction (López-Otín et al., 2023). Many of these processes overlap with ALS-related mechanisms (blue arcs) (Jagaraj et al., 2024) and with biological pathways associated with primary cilia (green arcs) (Silva and Cavadas, 2023). This convergence supports the emerging concept of ALS as a disorder that shares features with an “age-related ciliopathy.”

Lastly, it is important to understand the relationship between aging, primary cilia, and ALS. Although ALS can develop at a relatively young age and progresses much more rapidly than physiological aging, it typically manifests in midlife, and aging is recognized as a major risk factor for disease onset. Interestingly, nearly all of the 12 “hallmarks of aging” are closely involved in ALS pathophysiology, leading many researchers to propose that aging processes contribute to ALS onset and progression (Figure 3; López-Otín et al., 2023; Jagaraj et al., 2024). At the same time, as noted above, primary cilia are profoundly associated with aging, and the concept of “age-related ciliopathy” has recently attracted considerable attention. Considering ALS from the perspective of an age-related ciliopathy may therefore provide important new insights into its mechanisms.

In conclusion, research into primary cilia in ALS remains in its early stages. Numerous technical and experimental hurdles must be addressed to bridge the gap between the basic biology of cilia and its clinical translation. A multidisciplinary approach that integrates molecular biology, neuropathology, and clinical neurology will be essential to advance this promising field and develop effective cilia-based therapeutic strategies.

## Conclusion

Primary cilia have emerged as crucial regulators of diverse signaling pathways for cellular homeostasis, development, and tissue maintenance. Recent evidence suggests that dysfunction of these pathways may contribute significantly to the pathogenesis of ALS. In this review, we have synthesized current knowledge on the structural and functional changes of primary cilia in ALS, the roles of ALS-associated genes such as *NEK1*, *C21orf2*, and *C9orf72* in ciliogenesis, and the bidirectional relationships between ciliary dysfunction and known ALS pathomechanisms. Accumulating evidence warrant a conceptual shift in our understanding of ALS, positioning primary cilia as both potential mediators and modulators of disease progression. Further, primary cilia may serve not only as a novel pathophysiological hub, but also as a promising therapeutic target.

Despite these insights, many critical questions remain. Whether ciliary defects are primary events or secondary consequences in ALS pathology is still unclear. In addition, the extent to which ciliary dysfunction contributes to sporadic versus familial ALS needs further clarification. Technical challenges in assessing cilia in human tissues and the lack of reliable biomarkers also hinder progress in this field.

To fully elucidate the role of primary cilia in ALS, multidisciplinary research strategies are essential. Integrating molecular and cellular studies with human neuropathological and clinical data will be key to translating basic cilia biology into therapeutic and diagnostic applications. In so doing, the cilia-ALS axis may open new avenues for understanding disease onset, progression, and treatment for this currently incurable condition.
